# Global Muscle Coactivation of the Sound Limb in Gait of People with Transfemoral and Transtibial Amputation

**DOI:** 10.3390/s20092543

**Published:** 2020-04-29

**Authors:** Antonella Tatarelli, Mariano Serrao, Tiwana Varrecchia, Lorenzo Fiori, Francesco Draicchio, Alessio Silvetti, Silvia Conforto, Cristiano De Marchis, Alberto Ranavolo

**Affiliations:** 1 Department of Human Neurosciences, University of Rome Sapienza, 00185 Rome, Italy; 2 Department of Occupational and Environmental Medicine, Epidemiology and Hygiene, INAIL, Monte Porzio Catone, 00185 Rome, Italy; t.varrecchia@inail.it (T.V.); f.draicchio@inail.it (F.D.); al.silvetti@inail.it (A.S.); a.ranavolo@inail.it (A.R.); 3 Department of Medico-Surgical Sciences and Biotechnologies, University of Rome Sapienza, 04100 Latina, Italy; mariano.serrao@uniroma1.it; 4 Department of Physiology and Pharmacology, University of Rome Sapienza, 00185 Rome, Italy; lorenzo.fiori@uniroma1.it; 5 Department of Engineering, Roma TRE University, 00185 Rome, Italy; silvia.conforto@uniroma3.it (S.C.); cristiano.demarchis@uniroma3.it (C.D.M.)

**Keywords:** lower limb amputation, prosthetic gait, muscle coactivation, surface electromyography

## Abstract

The aim of this study was to analyze the effect of the level of amputation and various prosthetic devices on the muscle activation of the sound limb in people with unilateral transfemoral and transtibial amputation. We calculated the global coactivation of 12 muscles using the time-varying multimuscle coactivation function method in 37 subjects with unilateral transfemoral amputation (10, 16, and 11 with mechanical, electronic, and bionic prostheses, respectively), 11 subjects with transtibial amputation, and 22 healthy subjects representing the control group. The results highlighted that people with amputation had a global coactivation temporal profile similar to that of healthy subjects. However, amputation increased the level of the simultaneous activation of many muscles during the loading response and push-off phases of the gait cycle and decreased it in the midstance and swing subphases. This increased coactivation probably plays a role in prosthetic gait asymmetry and energy consumption. Furthermore, people with amputation and wearing electronic prosthesis showed lower global coactivation when compared with people wearing mechanical and bionic prostheses. These findings suggest that the global lower limb coactivation behavior can be a useful tool to analyze the motor control strategies adopted and the ability to adapt to the prosthetic device.

## 1. Introduction

Lower limb amputation leads to significant neural reorganization within the central nervous system (CNS) mostly due to the loss of the sensorimotor function caused by amputation [1,2]. People with amputation need considerable walking training [3] to adopt a series of compensatory motor strategies involving both prosthetic and sound limbs [4,5,6,7]. Gait kinematic and kinetic analyses documented an asymmetric gait characterized by the shortened stance, enlarged double support durations, and reduced knee flexion and hip extension ranges of motion (ROM) in the prosthetic limb [8,9,10,11,12] and enlarged stance duration [9,13], augmented hip and knee joint ROM [6,8,14], ankle joint power [15], and vertical ground reaction force [16,17] in the sound limb. This abnormal gait pattern is dependent on the amputation level, being more pronounced, and with greater mechanical work, in people with transfemoral amputation (TFA) when compared with people with transtibial amputation (TTA) [6,18]. Studies evaluating the activity of the final motor effectors (i.e., muscles) revealed a higher and longer compensatory activity of the residual muscles in the prosthetic limb [9,19] and an altered activation of all muscle synergies in the sound limb before and after the prosthetic heel strike [7]. Overall, kinematic, kinetic, and surface electromyography (sEMG) gait findings reflect the compensatory efforts developed by people with amputation to protect the soft tissues of the sound limb [4] and to deal with the new prosthetic limb condition [7]. These compensatory mechanisms in the sound limb consist of increasing muscle activation, spending more time on the ground [20,21,22], and developing a greater and longer force production [22,23,24]. However, over time, an asymmetric gait may prove damaging to individuals. One of the main objectives of the gait analysis studies in people with amputation should be to improve the development of new and ergonomic prostheses, as well as to improve the adaptation ability of the people to the most recent and technologically advanced prosthetic devices [25,26,27]. The ideal prosthetic device should allow people with amputation to maintain an effective and ecological gait function and, at the same time, minimize gait asymmetries and reduce the need for the compensatory activation of the muscles of the sound limb. Some previous studies have revealed a change in the force asymmetry rate across the gait cycle based on the type of prosthesis [22,28,29]. Recently, Varrecchia et al. [6] showed that people using the bionic prosthetic knee Genium exhibited a better gait performance compared with those using the mechanical prosthesis because they walked with a longer step length and greater hip and knee ROM in the sound limb. Results from a recent systematic review [30] confirmed that the bionic prosthetic knee Genium allows a more physiological and symmetric gait with reduced compensatory movement in the sound limb. However, no investigations on the effect of various prosthetic devices on the muscle activation of the sound limb have been undertaken so far. Such investigations are required to ascertain the amounts of compensation in the neuromuscular strategies adopted for different prostheses. 

Herein, we investigated the effect of three different types of prosthetic devices (mechanical, electronic, and bionic) on the activation of the sound limb muscles. We used the time-varying multimuscle coactivation function (TMCf) method [31,32], a compact indicator that allows one to understand the global strategy adopted by the central nervous system (CNS) in modulating the simultaneous activation/deactivation of many lower limb muscles during gait, irrespective of the magnitude of the single muscle activation, to the agonist–antagonist interaction at the single joint level and to the modular architecture. We hypothesized that people with amputation might show a compensatory increase in global muscle coactivation based on the level of amputation (TFA > TTA) and type of prosthesis (bionic) and correlated with gait asymmetry, gait performance, and energy consumption.

## 2. Materials and Methods

### 2.1. Subjects

Before starting experimental procedures, the minimum sample size of 9 was chosen based on statistics, in order to have adequate statistical power [33]. Particularly, 9 or more measurements are needed to have a confidence level of 85% that the real value is within ±15% of the measured value. The study sample was defined after verifying that all patients met the inclusion criteria, i.e., they wore their prostheses for at least two years and were able to walk independently on level surfaces without external aids. Patients with chronic diseases, complications, or cognitive disorders have not been included. In doing so, 48 subjects with lower-limb unilateral TFA and TTA, consequent to workplace traumatic accidents, were enrolled from the Rome branch of the Prosthetics Center of Italian Workers’ Compensation Authority (INAIL). 22 healthy subjects were enrolled as the control group (C) and were age-sex-speed matched with people with amputation. All healthy subjects included did not have pathologies that could influence the normal gait pattern. 

This study was classified as an observational study on the base of the definitions of the European Directive 2001/20/EC. The study protocol was approved by the local ethics committee (UP 00978_2020, Sapienza-University of Rome) and conformed to the guidelines of the Declaration of Helsinki. All participants provided written informed consent.

### 2.2. Experimental Procedure

**Figure 1:**
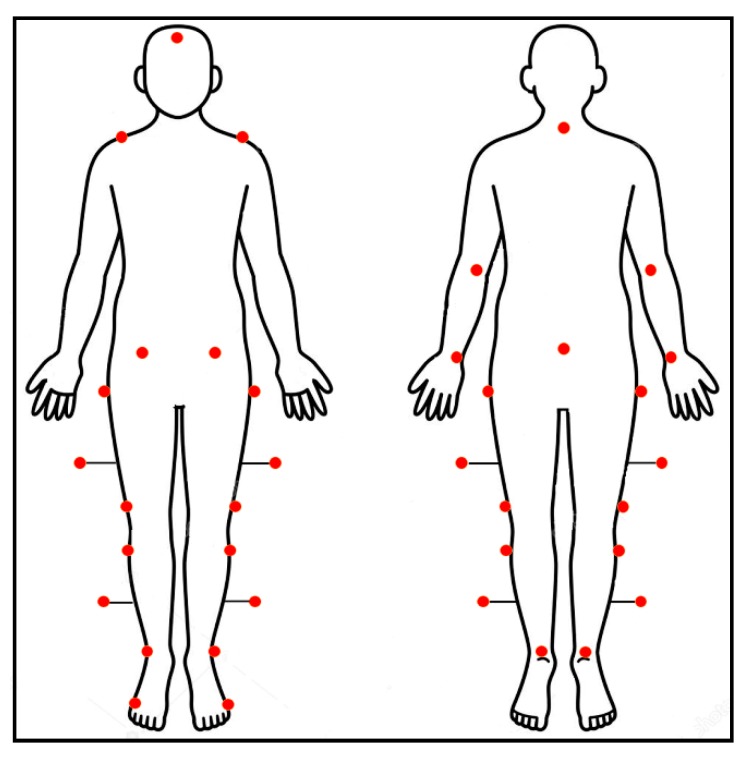
Modified Davis’ protocol for marker placement.

Walking tests were performed using a six infrared cameras optoelectronic motion analysis system at sample frequency of 340 Hz (SMART-DX 6000 System, BTS, Milan, Italy). Twenty-seven passive spherical markers were placed on the following prominent bony landmarks, according to a modified Davis’ protocol [32,34,35,36], as shown in Figure 1: the head, the 7th cervical vertebra (c7) and sacrum, bilaterally over the acromion, olecranon, ulnar styloid process, anterior superior iliac spine, great trochanter, lateral femoral condyle, fibula head, lateral malleoli, metatarsal head, and heel. Moreover, sticks markers were placed at 1/3 of the length of femur and leg segments:

After the placement of markers, anthropometric measurements were taken for each subject. Gait analysis started with the standing position on a platform. Subsequently, controls and subjects with amputation were asked to walk at their preferred speed with their shoes. Furthermore, controls were asked to walk also at a slower speed. At least ten trials, at each velocity, were recorded for both subject groups.

### 2.3. Data Analysis

For each acquisition, marker trajectories were reconstructed using a frame-by-frame tracking software (SMART Tracker, BTS, Milan, Italy). Data were processed using the SMART Analyzer (BTS, Milan, Italy) and Matlab software (version 7.10.0, MathWorks, Natick, MA, USA). 

#### 2.3.1. Matching Procedure

People with amputation and controls were matched for age, sex, and speed. In particular, only the walking trials of controls, at preferred or slower speeds, whose mean walking speed fell within the range identified with people with amputation mean walking speed ± SD [37,38] were considered. Subsequently, an unpaired two-sample t-test (statistical significance with *p*-value set at 0.05) was used to verify that the mean speed values were not statistically different between people with amputation and controls, both TFA vs. controls (C_TFA_) and TTA vs. controls (C_TTA_). 

#### 2.3.2. Electromyographic Data

We recorded sEMG signals using a bipolar 16-channel wireless system (FreeEMG 1000 System, BTS) with a sample frequency of 1000 Hz. Pairs of Ag/AgCl surface electrodes (distance between two electrode centers: 2 cm) were placed on the sound limb of the people with amputation and on the dominant side of the controls on the gluteus medius, rectus femoris, vastus lateralis, vastus medialis, tensor fascia latae, semitendinosus, biceps femoris, tibialis anterior, gastrocnemius medialis, gastrocnemius lateralis, soleus, and peroneus longus in accordance with *Atlas of Muscle Innervation Zones* [39] and the European Recommendations for Surface Electromyography [40]. Electromyographic data was interpolated to 201 samples [32,41,42,43] using a polynomial procedure time-normalizing to the duration of the gait cycle (time between two consecutive foot contacts of the same leg).

#### 2.3.3. Global Coactivation of Lower Limb Muscles

The raw sEMG signals were band-pass filtered (3rd order Butterworth filter at 30–450 Hz) and then rectified and low-pass filtered (zero-lag 4th order Butterworth filter at 10 Hz). For each individual, the sEMG signal from each muscle was normalized to its peak median value across all strides of all trials. From the processed sEMG signals, we evaluated the simultaneous activation of 12 lower limb muscles by considering the TMCf [31,32,41,44] calculated by the following equation:

(1) $$TMCf\left(d\left(i\right),i\right)=\left(1-\frac{1}{1+e^{-12\left(d\left(i\right)-0.5\right)}}\right).\frac{{\left(\sum_{h=1}^{H}EMG_{h}\left(i\right)/H\right)}^{2}}{max_{h=1\ldots H}\left[EMG_{h}\left(i\right)\right]}\;,$$

where *H* is the number of muscles considered, *EMG_h_(i)* is the sEMG sample value of the *h*th muscle at instant *i*, *d*(*i*) is the mean of the differences between each pair among the twelve *EMG_h_(i)* samples at instant *i*:

(2) $$d\left(i\right)=\left(\frac{\sum_{h=1}^{H-1}\sum_{n=h+1}^{H}\;|EMG_{h}\left(i\right)-EMG_{n}\left(i\right)|}{\left(\frac{H!}{2!\left(H-2\right)!}\right)}\right)\;,$$

where *H*!/(2!(*H*−2)!) is the total number of possible differences between each pair of *EMG_h_(i)*. Next, we calculated the coactivation index (CI) as the mean value of the TMCf [31]:

(3) $$CI=\overset{201}{\underset{i=1}{\sum }}\frac{TMCf\left(d\left(i\right),i\right)}{201}$$

##### Full Width at Half Maximum and Center of Activity

For both people with amputation and controls, we computed the full width at half maximum (FWHM_TMCf_) to characterize in terms of time amplitude the TMCf curves. The FWHM for each TMCf waveform was calculated as the sum of the durations of the intervals $\left(\Delta t_{j}\right)$ in which the TMCf curve exceeded half of its maximum:

(4) $$FWHM=\underset{j}{\sum }\Delta t_{j}$$

Furthermore, we computed the center of activity (CoA_TMCf_) to understand where most coactivation is concentrated within the gait cycle [42]. It was calculated by integrating the formula adopted by Labini [45] with the circular transformation, obtaining the following expression:

(5) $$coa=tan^{-1}\left(\frac{\sum_{i=0}^{201}\;EMG_{i}\times sin\vartheta_{i}}{\sum_{i=0}^{201}EMG_{i}\times \cos\vartheta_{i}\;}\right)\;,$$

where *θ_i_* is the *i*th sample of the 0%–100% gait cycle scale transformed into a 0–360° angular scale. 

##### Coefficient of Multiple Correlation

The waveform similarity of the curves was measured with the coefficient of multiple correlation (CMC): the closer to 1 this index is, the more similar the waveforms are [46,47]. In particular, we calculated:

- for each group, the within-subject similarity for TMCf (CMC_TMCf_IS_) among all TMCf curves of all strides for each subject and then, we computed the mean and standard deviation of the CMC_TMCf_IS_ of all subjects within each group;
- the between-subject similarity on the mean TMCf curves (CMC_TMCf_BS_) of all subjects of each group;
- the similarity among the mean TMCf curves of the three groups, evaluated among all the subjects (CMC_TMCf_BG_).

We calculated the coefficient of multiple correlation as follows:

(6) $$CMC=\sqrt{1-\frac{\left(1/\left(T\;\left(N-1\right)\right)\right)\sum_{n=1}^{N}\sum_{t=1}^{T}{\left(y_{nt}-{\bar{y}}_{t}\right)}^{2}}{\left(1/\;\left(T\;N-1\right)\right)\sum_{n=1}^{N}\sum_{t=1}^{T}{\left(y_{nt}-\bar{y}\right)}^{2}}}\;,$$

where *T* = 201 (number of time points within the cycle), *N* is the number of curves, $y_{nt}$ is the value at the *t*th time point in the *n*th curve, and ${\bar{y}}_{t}$ is the average at time point *t* over N curves:

(7) $${\bar{y}}_{t}=\frac{1}{N}\;\sum_{n=1}^{N}y_{nt},$$

where $\bar{y}$ is the grand mean of all$\;y_{nt}$:

(8) $$\bar{y}\;=\frac{1}{NT}\sum_{n=1}^{N}\sum_{t=1}^{T}y_{nt}$$

##### Deviation Phase

Deviation phase (DP) is calculated by averaging the standard deviations of the ensemble TMCf curves for each group using the following equation [46]:

(9) $$DP=\frac{\sum_{i=1}^{p}\;SD_{i}}{p}\;,$$

where *p* is the number of time points. 

#### 2.3.4. Time-Distance Parameters

The following time-distance parameters were calculated for each subject with amputation: walking speed (m/s), cadence (steps/s), step length (m), and step width (m) normalized to the limb length; stance, swing, and double support phase duration expressed as percentages of the gait cycle duration. 

##### Symmetry Index

Symmetry index was calculated for each time-distance parameter (*X*) using the following formula [48]:

(10) $$SI=\frac{\left(X_{P}-X_{SL}\right)}{0.5\times \left(X_{P}+X_{SL}\right)}\times 100,$$

where *X_P_* and *X_SL_* are the parameters for the prosthetic and sound limbs, respectively. The value *SI* = 0% indicates full symmetry, and *SI* = 100% indicates full asymmetry [49].

#### 2.3.5. Energy Expenditure Parameters

The mechanical behavior in terms of energy consumption (TEC) [50] and recovery (R-step) [51] was calculated in relation to the whole-body center of mass (CoM) kinematics during walking evaluated by means of the “reconstructed pelvis method” considering the kinematic data [52].

The kinetic energy (*E_k_*) associated with CoM displacements during the gait cycle was calculated as the sum of the kinetic energy on the x (*Ek_x_*), y (*Ek_y_*), and z (*Ek_z_*) axes as follows:

(11) $$E_{k}=E_{kx}+E_{ky}+E_{kz}=\frac{1}{2}m\left(v_{x}^{2}+v_{y}^{2}+v_{z}^{2}\right)$$

where *m* and *v_x_*, *v_y_*, and *v_z_* are the mass and velocity components of the CoM, respectively. Furthermore, the potential energy (*E_p_*) associated with the CoM was calculated as

(12) $$E_{p}=mgh$$

where *h* is the vertical component of the CoM, and g is the acceleration of gravity (m/$s^{2}$).

The total mechanical energy (*E_tot_*) associated with the CoM was computed as the sum of *E_k_* and *E_p_*.

We calculated the fraction of mechanical energy (R-step) recovered during each walking step [51], as follows:

(13) $$R-step=\frac{W_{p}^{+}+W_{kf}^{+}-W_{tot}^{+}\;}{W_{p}^{+}+W_{kf}^{+}}\times 100=\left(1-\frac{W_{tot}^{+}\;}{W_{p}^{+}+W_{kf}^{+}}\right)\times 100$$

where $W_{p}^{+},W_{kf}^{+},W_{tot}^{+}$ represent the positive work produced by the gravitational potential energy, kinetic energy of forward motion, and total mechanical energy, respectively. Then we calculated the total energy consumption (TEC) as the sum of the negative ($W_{tot}^{-}$) and positive work ($\;W_{tot}^{+}$), each divided by their respective efficiencies [53], as follows:

(14) $$TEC=\frac{W_{tot}^{+}\;}{1.20}+\frac{W_{tot}^{-}\;}{0.25}$$

Given the cyclic nature of walking, the positive work done at each step is equal to the negative work, which thus changes the previous formula to

(15) $$TEC=\frac{W_{tot}^{+}\;}{0.21}$$

For each subject, the R-step and TEC values were normalized to the body weight and step length, respectively, and were averaged.

### 2.4. Statistical Analysis

The Kolmogorov–Smirnov and Shapiro–Wilk tests were used to verify the normal distribution of the data. An unpaired two-sample t-test (the statistical significance was established for *p* < 0.05) was used to verify the presence of significant differences between TFA and C_TFA_ and between TTA and C_TTA_ for each EMG variable. 

A three-level one-way ANOVA was performed to evaluate the effect of the type of prosthesis (TFA_M_, TFA_C_, and TFA_G_) on each EMG variable. When relevant differences were observed in the ANOVA we performed post-hoc analyses with Bonferroni’s corrections. 

As regards the CoA, we performed the Watson–Williams test for circular data [54] to verify the presence of significant differences between TFA and C_TFA_, between TTA and C_TTA_, and among the three subgroups (TFA_M_, TFA_C_, and TFA_G_) of subjects with TFA (effect of the type of prosthesis). 

We used Pearson’s coefficient to investigate any correlation between TMCf indexes and time-distance parameters, symmetry indexes and energetic parameters. The correlation level was defined based on the following values [55]:

- weak correlation for 0 < *r* < 0.3;
- moderate correlation for 0.3 < *r* < 0.7;
- strong correlation for *r* > 0.7.

All analyses were performed using Matlab (version 7.10.0, MathWorks, Natick, MA, USA).

## 3. Results

### 3.1. People with Amputation

The study group included thirty-seven people with TFA (3 women and 34 men; age: 53.92 ± 14.13 year; weight: 82.18 ± 12.84 kg), that used mechanical [56,57] and two types of MPKs prosthesis i.e., Cleg and Genium (Ottobock, Duderstadt, Germany) [27] prostheses [6], and eleven people with TTA (age: 59.4 ± 12.8 year; weight: 85.08 ± 12.7 kg). Among people with TFA, 10 wore mechanical (TFA_M_), 16 CLeg (TFA_C_), and 11 Genium (TFA_G_) prostheses. TTA wore the same type prosthetic foot (Ossur Variflex foot, Ossur Reykjavík, Islanda [58]) but the sockets were custom-made and adapted to the patients’ anatomical characteristics and needs.

### 3.2. Lower Limb Global Coactivation

#### 3.2.1. People with Amputation versus Controls

**Figure 2:**
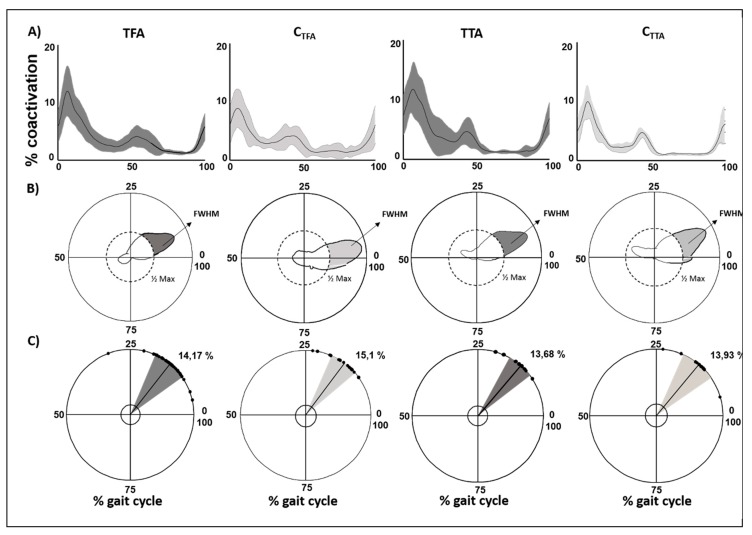
(**A**) Time-varying multimuscle coactivation function (TMCf) curves shown as mean curves with standard deviations for people with transfemoral amputation (TFA), the control group matched with TFA (C_TFA_), for people with transtibial amputation (TTA), and the control group matched with TTA (C_TTA_). (**B**) Full width at half maximum (FWHM) of the TMCf for each group shown in polar coordinates. (**C**) Center of activity (CoA) of the TMCf for each group: each dot represents the mean CoA value of a subject, whereas the solid line and the width of the circular sector represent the mean and standard deviation values of the CoA of all subjects, respectively. All parameters are shown as percentages of the gait cycle.

The global coactivation curves of the sound limb muscles of TFA and TTA groups and their respective matched controls are shown in Figure 2A. 

Both TFA and TTA groups showed significantly higher CI values when compared with the corresponding controls (Table 1). 

Both TFA and TTA groups showed significantly higher FWHM values when compared with the corresponding controls (TFA vs. C_TFA_: *p* = 0.03 and TTA vs. C_TTA_: *p* = 0.04). 

Both TFA and TTA groups showed significantly higher DP values than those of the corresponding controls (Table 1). 

No significant differences in the CMC_IS_ were found between the TFA and TTA groups when compared with the corresponding controls (Table 1). 

The level of amputation had no statistically significant effect on CoA evaluated on TMCf curves (*p*, all > 0.05).

#### 3.2.2. Type of Prosthesis

**Figure 3:**
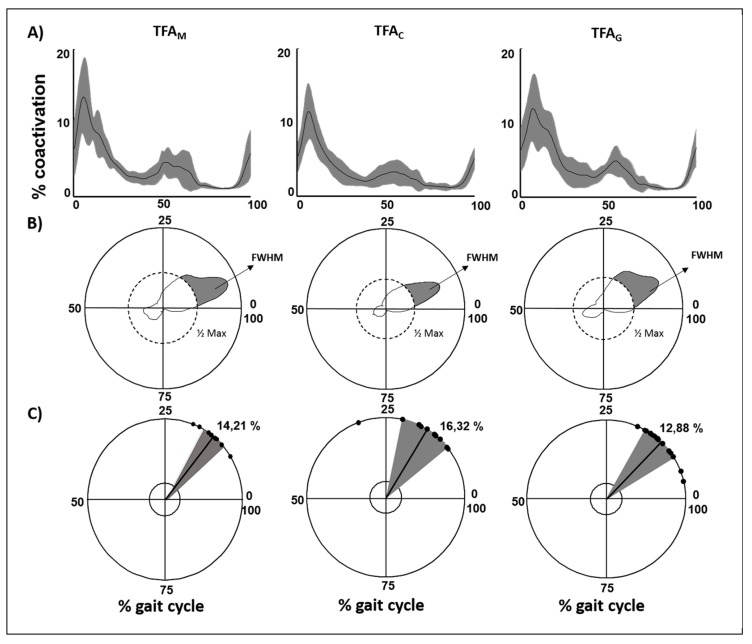
(**A**) TMCf curves shown as mean curves with standard deviations for people with transfemoral amputation with mechanical (TFA_M_), CLeg (TFA_C_), and Genium prostheses (TFA_G_). (**B**) Full width at half maximum (FWHM) of the TMCf for each group shown in polar coordinates. (**C**) Center of activity (CoA) of the TMCf for each group: each dot represents the mean CoA value of a subject, whereas the solid line and the width of the circular sector represent the mean and standard deviation values of the CoA of all subjects, respectively. All parameters are shown as percentages of the gait cycle.

Figure 3A shows the global coactivation for the three subgroups of patients with transfemoral amputation (TFA_M_, TFA_C_, and TFA_G_). 

A significant main effect of the type of prosthesis was found on the CI values. TFA_C_ showed lower values when compared with both TFA_M_ and TFA_G_ at post-hoc analysis (Table 1).

A significant main effect of the type of prosthesis was found on the FWHM values. TFA_C_ showed lower values when compared with both TFA_M_ and TFA_G_ at post-hoc analysis (*p* = 0.036).

A significant main effect of the type of prosthesis was found on DP values. TFA_C_ showed lower values when compared with TFA_M_ and TFA_G_ (Table 1).

No significant effect of the type of prosthesis was found on CMC_IS_ values (Table 1). 

The type of prosthesis had no statistically significant effect on CoA evaluated on TMCf curves (*p*, all > 0.05).

### 3.3. Correlation Finding

A moderate positive correlation was found between CI and gait speed values in people with TFA (*p* = 0.03, *r* = 0.36). Correcting for gait speed, partial correlation analysis showed a moderate positive correlation between CI and stance duration values (*p* = 0.04, *r* = 0.38). Furthermore, people with TFA also showed a moderate positive correlation between CI and TEC values (*p* = 0.04, *r* = 0.52) and a negative correlation between the DP and symmetry index evaluated on double support duration (*p* = 0.04, *r* = −0.33).

## 4. Discussion

This study investigated the effect of the level of amputation and the type of prosthesis on the global coactivation of the sound limb in people with amputation during walking. We used a time-varying function that allows the simultaneous study of the activation of many muscles [31] and can provide information on the global compensatory strategy adopted by people with amputation in the sound limb. The increased CI and FWHM values in the sound limb indicate the need for people with amputation to increase the level of the simultaneous activation of many muscles and for a longer time when compared with healthy controls. This is an expected result and well reflects the compensatory increase in stiffness, force production, and stance time in the sound limb demonstrated in several previous studies [6,59]. The characteristic double-peak shape curve of the coactivation function (Figure 2A), together with the lack of significant differences in the CoA values when compared with the control, suggests that the global coactivation temporal profile in people with amputation is similar to that of healthy subjects. In particular, we found that the global coactivation increased during the loading response (weight acceptance) and push-off phases of the gait cycle, which are the most challenging subphases in terms of force interaction between the foot and ground, and decreased in the mid-stance and swing subphases. This finding points out that, although increased, the motor control underlying the global coactivation is preserved in the sound limb of people with amputation and thus has a purely compensatory valence. This notion is further reinforced by the observation that the spatiotemporal modular architecture of the muscle synergies in the sound limb is preserved in people with TFA [7]. We found that in people with TFA, the global coactivation correlated positively with the stance duration and energy consumption and negatively with the swing duration and double-support gait asymmetry. The higher the global coactivation, the higher the energy consumption and the lower the gait asymmetry. This finding suggests that compensatory coactivation of the sound limb muscles may relevantly contribute to both the asymmetry and excessive energy expenditure, typically characterizing the prosthetic gait. We also found a significantly increased DP in both people with TTA and TFA when compared with controls, without any difference between them. The DP measure expresses the variance of the global coactivation curves and thus indicates an increased intersubject variation in the global coactivation function in people with amputation, which is not related to the level of amputation. This finding, in addition to the lack of significant difference in the intrasubject variability, as evaluated by CMC, suggests that the compensatory increase in global coactivation of the sound limb muscles is variably exerted by people with amputation, from subject to subject, but remains stable within each subject. We found a significant effect of the type of prosthesis, therefore it is likely that the intersubject global coactivation variability may depend on the type of prosthesis. An interesting finding of our study is that the type of prosthesis affected global coactivation. Among people with TFA, TFA_C_ showed significantly lower CI and DP values than both TFA_M_ and TFA_G_. The found differences could have been independent of the knee system but an expression of individual patient characteristics that lead to the differences in prescription. Although the difference between mechanical and electronic prosthetic devices is an expected result, in line with a previous study on kinematic and kinetic data [60,61], the difference between electronic (C-Leg) and bionic (Genium) devices, with lower values of CI and DP in the former, is an unexpected result. Previous studies [27,62] have shown that TFA_G_ showed improved upper body flexibility, balance, and endurance when compared with TFA_C_ during either walking or climbing stairs. These previous observations suggest that there is less need to coactivate sound limb muscles in TFA_G_ even more than in TFA_C_. A possible explanation for our finding might be attributed to the observed greater knee flexion of the prosthetic limb in TFA_G_ when compared with TFA_C_ [6,27,62]. The increased knee flexion yields an increased swing duration in the prosthetic limb, which, in turn, may imply the need to maintain a compensatory higher level of global coactivation in the sound limb of TFA_G_ than in TFA_C_ This greater muscle coactivation could have a multifactorial interpretation: the Genium device, offering the possibility to program more activities, could lose performance on specific tasks. Two other factors derive from the technical characteristics of the device: the greater weight of the prosthesis and the introduction of a knee pre-flexion in the double-support phase, which is often not well managed by the patient. However, these results should be taken with caution considering the small sample size of subjects when grouping people with amputation based on the type of prosthesis. Further studies on larger samples are warranted to specifically investigate the differences in muscle activation between subjects with C-Leg and Genium devices.

The global coactivation measure may represent a useful tool to characterize motor control and adaptation to the prosthesis. Furthermore, it can be used to identify the most efficient rehabilitation treatment over time. 

## 5. Conclusions

Our findings demonstrate that people with amputation increase the muscle coactivation of the sound limb during walking as a compensatory/adaptive mechanism to deal with the prosthetic device. This increased coactivation probably plays a role in prosthetic gait asymmetry and energy consumption. TFA_C_ showed lower global coactivation values when compared with TFA_M_ and TFA_G._

## Figures and Tables

**Table 1:** The means, standard deviations, and statistical results (*p*-values) of parameters evaluated on TMCf curves (CI: coactivation index, CMC_IS_: coefficient of multiple correlation intra-subjects, DP: deviation phase). People with transfemoral amputation (TFA), control group matched with TFA (C_TFA_), people with transtibial amputation (TTA), control group matched with TTA (C_TTA_), people with transfemoral amputation with mechanical (TFA_M_), CLeg (TFA_C_), and Genium prostheses (TFA_G_).

**People with Amputation versus Controls**							**Type of Prosthesis**			
	**TFA**	**C_TFA_**	**p_group_**	**TTA**	**C_TTA_**	**p_group_**	**TFA_M_**	**TFA_C_**	**TFA_G_**	**p_group_**
**CI**	3.06 ± 0.58	2.43 ± 0.57	<0.01	3.11 ± 1.07	2.25 ± 0.31	<0.01	3.36 ± 0.55	2.78 ± 0.58	3.24 ± 0.48	0.02
**CMC_IS_**	0.84 ± 0.06	0.85 ± 0.05	>0.05	0.88 ± 0.04	0.88 ± 0.04	>0.05	0.86 ± 0.06	0.84 ± 0.05	0.84 ± 0.06	>0.05
**DP**	1.36 ± 0.31	1.07 ± 0.3	<0.01	1.28 ± 0.41	0.9 ± 0.2	0.01	1.43 ± 0.28	1.21 ± 0.26	1.41 ± 0.21	0.04
